# Combination therapy of pasireotide and pegvisomant for aggressive acromegaly with an immature PIT1-lineage PitNET

**DOI:** 10.1530/EDM-25-0171

**Published:** 2026-06-17

**Authors:** Keiko Tomiyama, Izumi Fukuda, Shigeyuki Tahara, Chie Inomoto, Robert Yoshiyuki Osamura, Masato Iwabu

**Affiliations:** ^1^ Department of Endocrinology, Metabolism and Nephrology, Graduate School of Medicine, Nippon Medical School, Bunkyo, Tokyo, Japan; ^2^ Department of Neurological Surgery, Nippon Medical School Musashikosugi Hospital, Kawasaki, Kanagawa, Japan; ^3^ Department of Pathology, Tokai University Hachioji Hospital, Hachioji, Tokyo, Japan; ^4^ Department of Pathology, Nippon Kokan Hospital, Kawasaki, Kanagawa, Japan

**Keywords:** pituitary, rare disease/syndromes

## Abstract

**Summary:**

A 29-year-old male presented with central scotoma and was suspected of having acromegaly based on enlargement of facial features and extremities. Serum growth hormone (GH) and insulin-like growth factor 1 (IGF-1) levels were elevated to 10.14 ng/mL and 571 ng/mL (74.8 nmol/L, +5.73 SDS), respectively. Magnetic resonance imaging revealed a giant pituitary neuroendocrine tumor (PitNET) measuring 48 × 48 × 58 mm with bilateral cavernous sinus invasion and high T2-weighted signal intensity. Because of poor GH suppression during an acute octreotide test (100 μg subcutaneously; nadir GH 6.25 ng/mL, 19% reduction from baseline) and the invasive tumor phenotype, preoperative pasireotide (PAS) 40 mg every 4 weeks was initiated. After 2 months, partial tumor removal was performed via transsphenoidal surgery. Histological analysis revealed an immature PIT1-lineage PitNET with strong membranous expression of somatostatin receptor subtypes 2 and 5. Persistently elevated IGF-1 after surgery led to re-initiation of PAS (40 mg every 4 weeks, escalated to 60 mg), followed by the addition of pegvisomant (PEG) 10 mg/day because biochemical control could not be achieved. Combination therapy with PAS and PEG normalized IGF-1 and maintained radiological tumor stability during long-term follow-up. This case highlights that biochemical response to somatostatin receptor ligands does not necessarily correlate with receptor expression in invasive PitNETs. Pathological classification using transcription factors may help guide individualized treatment strategies. Combination therapy with PAS and PEG may represent an effective option for aggressive acromegaly caused by immature PIT1-lineage PitNETs.

**Learning points:**

- Immature PIT1-lineage PitNETs may demonstrate discordance between somatostatin receptor expression and clinical responsiveness to somatostatin receptor ligands.
- In patients with predictors of poor response to first-generation somatostatin analogs, such as T2-weighted MRI hyperintensity and poor GH suppression during acute octreotide testing, early consideration of pasireotide (PAS) or combination therapy with PAS and pegvisomant (PEG) may be appropriate.
- In this case, PAS up to 60 mg every 4 weeks failed to adequately control IGF-1 levels, whereas subsequent combination therapy with PAS and PEG achieved biochemical remission and tumor stabilization, highlighting the potential role of combination strategies in aggressive acromegaly.

## Background

The immature PIT1-lineage pituitary neuroendocrine tumor (PitNET) is a rare subtype, accounting for approximately 0.9% of all PitNETs (1). Previously referred to as ‘silent subtype 3 adenoma’ or ‘PIT1-positive plurihormonal pituitary adenoma’, this subtype was newly defined in the 2022 WHO classification (2). Most immature PIT1-lineage PitNETs are clinically nonfunctioning, although a small proportion present with acromegaly, and tend to exhibit aggressive biological behavior (2).

Surgery is the primary treatment for acromegaly; however, remission rates are significantly lower for invasive PitNETs. Nevertheless, optimal treatment strategies for aggressive PitNETs that cannot be cured by surgery remain uncertain.

We present a case of aggressive acromegaly caused by an immature PIT1-lineage PitNET that exhibited poor biochemical response to pasireotide (PAS) monotherapy, despite strong somatostatin receptor (SSTR) 2 and SSTR5 expression, but responded to a combination therapy of PAS and pegvisomant (PEG).

## Case presentation

A 29-year-old man visited an ophthalmologist with a chief complaint of decreased vision. Visual field examination revealed central scotoma. He did not report headaches or other neurological symptoms. Physical examination revealed acromegalic features, including enlargement of the nose, hands, and feet, thickening of the lips, and prognathism. His medical history was unremarkable, and there was no family history of endocrine disorders. The patient was 172 cm tall with a normal growth pattern and weighed 65 kg.

## Investigation

Laboratory tests revealed elevated GH (10.14 ng/mL) and IGF-1 (571 ng/mL (74.8 nmol/L), reference range: 111–309 ng/mL (14.5–40.4 nmol/L), +5.73 standard deviation score (SDS)). The diagnosis of acromegaly was confirmed based on the elevated IGF-1 levels and unsuppressed GH during a 75 g oral glucose tolerance test (75 g OGTT) (GH-nadir: 9.42 ng/mL) (Table 1A). An acute octreotide suppression test was performed using 100 μg subcutaneous octreotide, with GH measurements obtained at baseline and every 2 h up to 8 h after administration. In the octreotide acute stimulation test, a minimum GH level of approximately 2.6 ng/mL or higher was defined as a poor response (3). Serum GH was measured using an electrochemiluminescence immunoassay (E-test ‘TOSOH’ II HGH; Tosoh Corporation, Japan). IGF-1 was not assessed in parallel during this test. GH showed only minimal suppression, with a nadir of 6.25 ng/mL at 2 h (19% reduction from baseline) (Table 1B). A bromocriptine test (2.5 mg orally) similarly failed to suppress GH (nadir 10.31 ng/mL) (Table 1C).

**Table 1:** Plasma GH responses to the 75 g oral glucose tolerance test (A), octreotide (B), and bromocriptine test (C).

	0 min	30 min	60 min	90 min	2 h	4 h	6 h	8 h
A. 75 g oral glucose tolerance test								
GH (ng/mL)	9.24	9.87	9.42	9.56	9.48			
Plasma glucose (mg/dL)	98	169	190	156	141			
Insulin (μIU/mL)	17.0	128.6	154	118.3	129.5			
B. Octreotide test (100 μg s.c.)								
GH (ng/mL)	7.68				6.25	8.01	10.26	10.12
C. Bromocriptine test (2.5 mg p.o.)								
GH (ng/mL)	11.07				10.73	10.31	11.33	12.31
PRL (ng/mL)	20.7				9.0	6.3	5.4	5.8

GH, growth hormone; PRL, prolactin.

**Figure 1:**
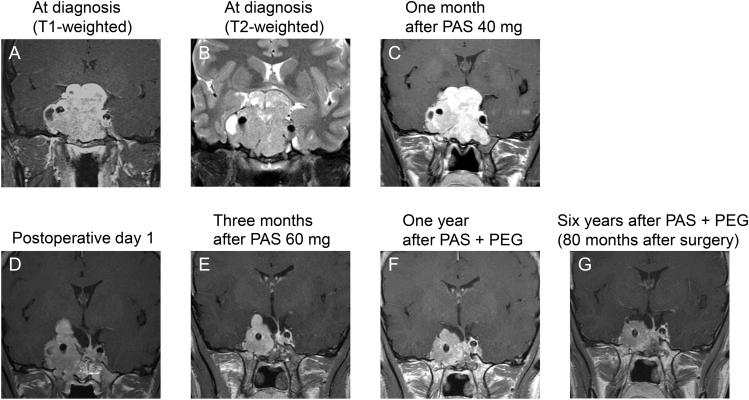
Serial pituitary MRI during the clinical course. (A) Coronal contrast-enhanced T1-weighted MRI at diagnosis showing a giant PitNET measuring 48 × 48 × 58 mm with suprasellar extension and bilateral cavernous sinus invasion (Knosp grade 4). (B) Coronal T2-weighted MRI demonstrating high signal intensity. (C) MRI after 1 month of preoperative pasireotide (PAS) therapy, showing modest tumor reduction (48 × 46 × 51 mm). (D) Early postoperative MRI demonstrating residual tumor (28 × 46 × 51 mm) invading the cavernous sinus. (E) MRI 3 months after PAS 60 mg, showing further reduction (25 × 36 × 51 mm). (F) MRI 1 year after PAS–pegvisomant (PEG) combination therapy, showing stable tumor size (26 × 31 × 48 mm). (G) MRI 6 years after PAS + PEG (80 months after surgery) demonstrating long-term tumor stability (26 × 29 × 42 mm).

Baseline pituitary hormonal testing showed elevated PRL (21.0 ng/mL, reference range: 3.0–17.3 ng/mL) and decreased luteinizing hormone (LH) (0.5 mIU/mL, reference range: 1.7–11.2 mIU/mL), follicle-stimulating hormone (FSH) (2.7 mIU/mL, reference range: 2.1–18.6 mIU/mL), and total testosterone (0.47 ng/mL, reference range: 1.31–8.71 ng/mL). ACTH, cortisol, and thyroid function were maintained. Pituitary magnetic resonance imaging (MRI) exhibited a giant PitNET (48 × 48 × 58 mm) compressing the optic chiasma and invading the bilateral cavernous sinuses (Fig. 1A and B), with high signal intensity on T2-weighted images. The 75 g OGTT also showed impaired glucose tolerance (Table 1A), and HbA1c was 5.9%. Hyperphosphatemia and hypertriglyceridemia were also noted.

Screening for acromegaly-related comorbidities was performed. Dual-energy X-ray absorptiometry showed normal lumbar spine bone mineral density (1.167 g/cm^2^; Z-score −0.4). Colonoscopy revealed no colorectal polyps or neoplasia. Thyroid ultrasonography demonstrated no thyroid nodules or malignancy.

## Treatment

The PitNET was invasive, extending into the cavernous sinus (Knosp grade 4), making complete surgical resection challenging. The high T2-weighted signal intensity on MRI and poor suppression during the octreotide test suggested limited responsiveness to first-generation somatostatin receptor ligands (SRLs).

**Figure 2:**
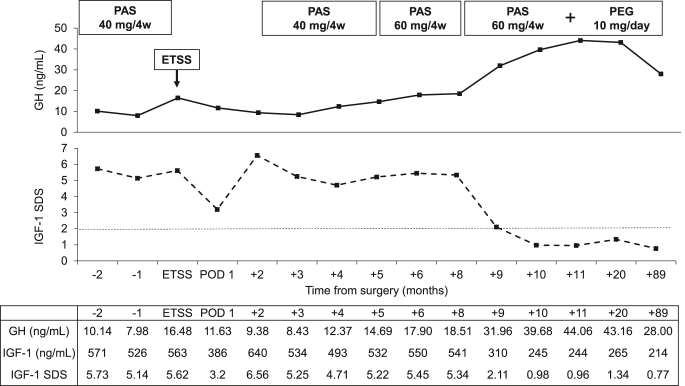
Serum GH and IGF-1 levels before and after surgery and during medical treatment. Preoperative PAS therapy produced only modest biochemical effects. After surgery, PAS was reinitiated and escalated to 60 mg every 4 weeks but failed to normalize IGF-1. PEG was subsequently added, resulting in normalization of IGF-1 levels. Because PEG interferes with GH immunoassays (E-test ‘TOSOH’ II HGH; Tosoh Corporation, Japan), treatment efficacy after PEG initiation was assessed primarily using IGF-1 concentrations. GH, growth hormone; IGF-1, insulin-like growth factor 1; ETSS, endoscopic transsphenoidal surgery; PAS, pasireotide; PEG, pegvisomant.

Therefore, preoperative PAS 40 mg every 4 weeks was initiated for 2 months to reduce tumor volume. After 1 month of PAS, MRI demonstrated a modest reduction in tumor size with the maximum diameter decreasing from 58 mm at diagnosis to 51 mm (Fig. 1C). GH decreased to 7.98 ng/mL and IGF-1 to 526 ng/mL (68.7 nmol/L, +5.14 SDS). However, after 2 months, GH increased to 16.48 ng/mL, and IGF-1 increased to 563 ng/mL (73.6 nmol/L, +5.62 SDS), corresponding to only a 1.4% reduction in IGF-1 from baseline and failure to achieve biochemical control (Fig. 2).

Endoscopic transsphenoidal surgery was subsequently performed to remove the tumor as extensively as possible, leaving the portion invading the cavernous sinus. Postoperative MRI confirmed residual PitNET measuring 28 × 48 × 51 mm with persistent cavernous sinus invasion (Fig. 1D).

## Outcome and follow-up

**Figure 3:**
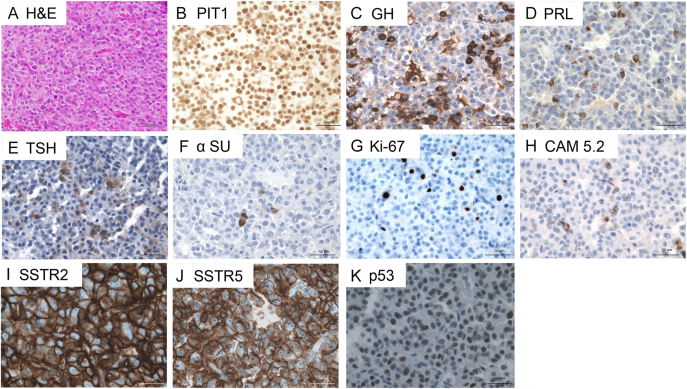
Histopathological findings of the pituitary tumor. (A) Hematoxylin–eosin staining showing an immature PIT1-lineage PitNET. (B) Tumor cells demonstrated diffuse nuclear PIT1 positivity, (C) diffuse GH positivity (∼30% of cells), (D) focal PRL (∼10%), (E) TSH (∼10%) positivity, and (F) rare α-subunit positivity (<5%). (G) The Ki-67 labeling index was 3.5%. (H) Cytokeratin CAM 5.2 staining was rarely positive without a dot-like pattern. (I, J) Somatostatin receptor immunohistochemistry revealed strong circumferential membranous expression of SSTR2 and SSTR5, each scored as 3+. (K) p53 immunostaining showing strong nuclear positivity. Scale bar = 50 μm. PIT1, pituitary-specific positive transcription factor 1; GH, growth hormone; PRL, prolactin; TSH, thyroid-stimulating hormone; *α*SU, *α*-subunit; SSTR, somatostatin receptor; CAM 5.2, cytokeratin.

Histological examination confirmed an immature PIT1-lineage PitNET with diffuse nuclear PIT1 positivity. Tumor cells showed diffuse GH positivity in approximately 30% of cells, focal PRL and TSH positivity in approximately 10% each, and rare *α*-subunit positivity (<5%). The Ki-67 labeling index was 3.5%, and the mitotic count was negligible. Cytokeratin (CAM 5.2) staining was rarely positive without a dot-like pattern. Immunohistochemical staining for p53 demonstrated strong nuclear positivity. In contrast, E-cadherin expression was negative. Semi-quantitative immunohistochemical evaluation demonstrated strong membranous expression of both SSTR2 and SSTR5 (score 3+) (Fig. 3).

Following surgery, GH and IGF-1 decreased transiently (postoperative day 1: GH 11.63 ng/mL; IGF-1 386 ng/mL (50.5 nmol/L, +3.20 SDS)) but subsequently increased. PAS was reinitiated at 40 mg every 4 weeks at 2 months postoperatively. GH and IGF-1 remained elevated (GH 14.69 ng/mL; IGF-1 532 ng/mL (69.6 nmol/L, +5.22 SDS)), prompting dose escalation to 60 mg every 4 weeks at 5 months postoperatively. Despite dose escalation, IGF-1 remained above the age-adjusted reference range (541 ng/mL (70.7 nmol/L, +5.34 SDS)), confirming poor biochemical response to PAS monotherapy (Fig. 2).

Eight months postoperatively, PEG was initiated with a loading dose of 40 mg followed by 10 mg daily. Treatment response was monitored using IGF-1 levels because PEG interferes with GH immunoassays. IGF-1 normalized within 2 months after PEG initiation (245 ng/mL (32.0 nmol/L, +0.98 SDS)) and remained within the age-adjusted reference range throughout follow-up (Fig. 2). Liver function tests were monitored monthly and remained within the normal range.

Regarding tumor size, the maximum tumor diameter stabilized at 51 mm after resumption of PAS (Fig. 1E) and measured 48 mm 1 year after initiation of combination therapy with PAS + PEG (Fig. 1F). At the latest follow-up, 80 months after surgery, MRI demonstrated radiological stabilization with slight reduction (Fig. 1G). Cavernous sinus invasion remained unchanged throughout follow-up, and additional radiotherapy was not required.

Pituitary function improved after surgery. Preoperative hypogonadism resolved, and testosterone increased from 0.47 ng/mL to 1.44 ng/mL. ACTH and cortisol levels normalized by 2 months postoperatively. No hormone replacement therapy was required, and diabetes insipidus did not occur.

After PAS initiation, fasting plasma glucose increased from 97 mg/dL to 126 mg/dL, and HbA1c increased from 5.8 to 6.0%. After PAS escalation to 60 mg, fasting plasma glucose increased to 174 mg/dL and HbA1c to 6.6%. Following PEG addition, glycemic control improved, with fasting plasma glucose decreasing to 112 mg/dL and HbA1c to 6.4% at 3 months and stabilizing at 6.3% after 1 year. No antidiabetic medications were required.

## Discussion

This case highlights the complexity of medical management in aggressive PitNET subtypes and underscores the importance of integrating pathological classification with clinical and imaging features when selecting therapeutic strategies.

SRLs remain central to the management of acromegaly. First-generation SRLs primarily target SSTR2, whereas PAS binds multiple receptor subtypes with a particularly high affinity for SSTR5 (4). A poor response to first-generation SRLs has been associated with several factors, including low SSTR2 expression, high Ki-67 index, young age, male sex, AIP mutations, low E-cadherin expression, high T2-weighted signal intensity, sparsely granulated histology, and poor GH suppression during acute octreotide testing (3, 5). Our patient exhibited several of these predictors, and immunohistochemistry demonstrated strong membranous expression of both SSTR2 and SSTR5. However, PAS monotherapy failed to normalize IGF-1 in our case. Even among SSTR5-positive PitNETs, a small subset has been reported to show limited responsiveness to PAS (6). This highlights that the absence of receptor expression strongly predicts non-responsiveness, whereas receptor positivity does not guarantee therapeutic efficacy. This is because treatment response is influenced not only by receptor expression but also by post-receptor signaling pathways, tumor granulation pattern, molecular background, and tumor lineage (5).

In patients with multiple predictors of resistance to first-generation SRLs, prolonged trials of first-generation SRLs may delay effective disease control. Earlier consideration of alternative strategies may be appropriate. In our patient, preoperative PAS therapy only modestly reduced the tumor size and failed to achieve biochemical control, leading to the addition of PEG.

PEG acts as a GH receptor antagonist and effectively lowers IGF-1 but does not directly inhibit tumor growth. Tumor enlargement has been reported in approximately 5% of PEG-treated patients, likely reflecting natural tumor progression (7). Given the invasive phenotype in our patient, concern existed regarding potential tumor progression under PEG monotherapy. Therefore, PAS was continued in combination with PEG to maintain tumor stabilization. This combined strategy resulted in biochemical remission and long-term radiological stability on serial MRI over long-term follow-up.

As a limitation, in this case, neither germline genetic testing nor immunohistochemical assessment of AIP expression was conducted. Germline AIP mutations or reduced AIP expression have been associated with resistance to first-generation SRLs (8, 9). Given our patient’s young age, tumor invasiveness, and suboptimal response to somatostatin analogs, evaluation of AIP status could have provided additional insight into the mechanisms underlying treatment resistance. Other hereditary causes of early-onset pituitary tumors, including MEN1, were also not investigated.

In addition to receptor-response mismatch, this combination strategy also has significant metabolic implications. While PAS can worsen glucose metabolism through inhibition of insulin and incretin hormones, combination therapy with PAS and PEG has been reported to mitigate hyperglycemia in some patients (10). Consistent with previous observations, HbA1c increased during PAS monotherapy but improved after initiation of combination therapy, stabilizing at approximately 6.2% with only dietary management.

In conclusion, immature PIT1-lineage PitNETs represent an aggressive subtype in which SSTR expression does not necessarily predict clinical responsiveness. This case emphasizes that strong SSTR2 and SSTR5 expression does not guarantee biochemical control with PAS. Pathological classification using transcription factors, together with imaging and biochemical characteristics, is essential for individualized treatment planning. Combined PAS and PEG therapy may represent a viable strategy for achieving durable biochemical control and tumor stabilization in aggressive acromegaly.

## Declaration of interest

The authors declare that there is no conflict of interest that could be perceived as prejudicing the impartiality of the research reported.

## Funding

This research did not receive any specific grant from any funding agency in the public, commercial, or not-for-profit sector.

## Patient consent

Written informed consent for publication of their clinical details and/or clinical images was obtained from the patient.

## Author contribution statement

All authors contributed individually to authorship. KT and IF were involved in the diagnosis, management, and manuscript submission. ST is a neurosurgeon who performed the surgery and has managed this patient during the course of his treatment. CI and RYO performed pathological analyses, including additional immunohistochemical studies, and contributed to the interpretation of pathological findings. MI undertook a critical review of the paper. All authors reviewed and approved the final version of the manuscript.
